# Involvement of the Gut–Lung Axis in LMW-PAHs-Induced Pulmonary Inflammation

**DOI:** 10.3390/toxics13121017

**Published:** 2025-11-25

**Authors:** Jiali Qin, Shiyao Jiang, Zhengyi Zhang, Jianding Wang, Yuanjie Li, Yunting Li, Haojun Zhang, Chengyun Li, Haitao Ma, Junling Wang

**Affiliations:** 1 Department of Toxicology, School of Public Health, Lanzhou University, Lanzhou 730030, China; 220220913580@lzu.edu.cn (J.Q.);; 2 School of Public Health, Southeast University, Nanjing 210009, China; 3 The Second Hospital & Clinical Medical School, Lanzhou University, Lanzhou 730030, China; zhangzhengyi11@lzu.edu.cn; 4 The Second Provincial People’s Hospital of Gansu, Lanzhou 730030, China

**Keywords:** low molecular weight PAHs (LMW-PAHs), gut microbiome (GM), gut–lung axis, lung inflammation, arachidonic acid (AA) metabolism

## Abstract

Polycyclic aromatic hydrocarbons (PAHs) are ubiquitous environmental pollutants recognized for their toxicological significance. Increasing evidence suggests that chronic exposure to low-molecular-weight PAHs (LMW-PAHs) contributes to heightened disease vulnerability and immune dysregulation, particularly among rural female populations. Recent studies have further linked a significant association between PAH exposure and gut microbiome (GM) modifications. Considering the common embryonic origin of the intestinal and respiratory systems, cross-organ communication under conditions of PAH exposure warrants deeper exploration. Although current gut–lung axis research largely emphasizes microbial metabolites such as short-chain fatty acids and bile acids, the contribution of arachidonic acid (AA) metabolites in LMW-PAH-induced pulmonary inflammation via this axis remains poorly defined. To address this knowledge gap, we developed an animal model employing integrated 16S rRNA sequencing and metabolomics approaches to systematically examine phenanthrene (Phe) and fluorene (Flu) induced GM compositional shifts and associated metabolic reprogramming. Through comprehensive profiling, we identified candidate microorganisms and metabolites potentially involved in dysbiosis-mediated pulmonary inflammation, thereby elucidating the mechanistic basis of Phe and Flu-associated health risks.

## 1. Introduction

Polycyclic aromatic hydrocarbons (PAHs) are a class of aromatic compounds with two or more fused benzene rings, widely distributed in environmental matrices like combustion-derived particulate matter and tobacco smoke. They pose significant toxicity and carcinogenic risks through multiple human exposure pathways [1,2]. PAHs primarily originating from anthropogenic activities with minor contributions from natural sources, present a range of health hazards [3,4]. Short-term PAH exposure worsens asthma and impairs lung function in at-risk individuals, increasing lung cancer risk. Chronic low-dose exposure causes sustained harm, including atherosclerosis, immune dysfunction, asthma progression, cardiopulmonary comorbidities, and reproductive issues [5,6]. PAHs can be categorized into low-molecular-weight PAHs (LMW-PAHs, containing 2–4 benzene rings) and high-molecular-weight PAHs (HMW-PAHs, containing more than 4 benzene rings) based on their molecular weight, which is primarily determined by the number of fused benzene rings. Although LMW-PAHs generally represent a larger proportion of total environmental PAHs relative to HMW-PAHs, they are generally regarded as posing comparatively lower immunological risks [7,8]. However, emerging in vivo and in vitro evidence shows LMW-PAHs cause inflammation and may be carcinogenic [9,10,11,12].

During the winter and spring heating seasons, the combustion of coal and biomass fuels for residential heating elevates the concentration of PAHs. Yao [13] reported that indoor PAH concentrations in rural northwestern China were significantly higher during the heating season compared to the non-heating period. Among the 16 priority-controlled PAHs, LMW-PAHs, including phenanthrene (Phe) and fluoranthene (Flu), demonstrated the highest concentrations. Specifically, the mean Phe concentration during the heating season reached 133.93 ng/m^3^, while that of Flu concentration was 83.58 ng/m^3^ [14,15]. An investigation of indoor dust across 28 Chinese provinces identified LMW-PAHs concentrated in northwestern and southern regions, with Phe (705 ng/g) and Flu (217 ng/g) as the predominant compounds [16]. LMW-PAHs (such as Phe, Flu) are known to trigger oxidative stress and inflammation in both animal models and cellular systems [10,17]. Although existing research has demonstrated the pro-inflammatory impact of LMW-PAHs on pulmonary tissues, the underlying mechanisms through which they modulate the pulmonary immune microenvironment remain incompletely elucidated.

For the majority of non-smoking individuals and those without occupational exposure, gastrointestinal ingestion represents the principal and most direct pathway for PAH exposure [18]. Inhalation exposure has been determined to contribute only 2–12% of total PAH exposure, whereas dietary intake accounts for the predominant 88–98% [19,20]. The dietary and inhaled total PAHs exposure of Beijing residents has been modeled, indicating that LMW-PAHs, such as Phe and Flu, account for approximately 85% of the population’s total PAH exposure [21]. The detection of PAHs in soil, dust, food, and drinking water across three districts of Lanzhou City, during both the heating and non-heating seasons, revealed that dietary exposure contributed to 33% of the total PAH intake. Notably, the carcinogenic risk associated with excessive PAH ingestion (from dust, food, and drinking water) was substantially higher than that of other exposure pathways [4]. Dietary PAHs pose greater health risks by disrupting the intestinal mucosal barrier, altering gut microbiota composition and abundance, and inducing intestinal inflammation [22,23,24,25]. The aforementioned studies have demonstrated that PAH exposure induces intestinal microbiota dysbiosis, characterized by an increased abundance of pathogenic bacteria and a concomitant suppression of beneficial bacterial populations.

Immune homeostasis relies on a microbiome-metabolite balance, while the pulmonary immune system maintains a labile equilibrium that dynamically interacts with microbiota due to aging, genetics, and environment [26,27]. Gut microbiota alterations, such as dysbiosis characterized by taxonomic shifts, reduced diversity, and impaired function, influence pulmonary disease pathogenesis by modulating lung immunity. The lung microbiota is also affected by gut-derived microbial signals [28]. As research on gut-respiratory crosstalk, the gut–lung axis hypothesis gains support, revealing mechanisms through which gut microbiota modulate pulmonary immunity and respiratory health [29]. Growing evidence shows gut–lung microbiota and metabolites drive axis interactions and influence pulmonary disease pathogenesis [30].

The current understanding of the gut–lung axis primarily focuses on short-chain fatty acids, bile acids, small organic amines, polyamines, vitamins, and other bioactive compounds. Nevertheless, the precise impact of PAHs on the gut–lung microbiota and the intermediary factors mediating gut–lung crosstalk remain poorly understood. Consequently, further investigation is warranted to elucidate the impact of gut–lung microbiota interactions on the pulmonary microenvironment from this perspective. Key microbial taxa and their metabolites may represent promising targets for early prevention and therapeutic intervention in lung diseases. This study employed an integrated approach of 16S rRNA sequencing and metabolomics to investigate the relationship between pulmonary inflammation induced by Phe and Flu via gut microbiota modulation. The objectives were to identify potential bioactive metabolites and elucidate their underlying mechanisms of action.

## 2. Materials and Methods

### 2.1. Experimental Animals and Chemicals

Thirty-two female Wistar rats, aged 6–8 weeks and weighing 160–190 g, were procured from Lanzhou Veterinary Research Institute, Chinese Academy of Agricultural Sciences. Following a week of acclimation in a controlled environment at 22–25 °C with 45–55% humidity and a 12 h light–dark cycle, the bedding in their cages was changed twice weekly. Following the animals’ acclimatization, they were randomly assigned to four groups (*n* = 8 per group) using a random number table. Since Phe (purity 98%, P11409, SIGMA, St. Louis, MO, USA) and Flu (purity 98%, 128333, SIGMA) are insoluble in water but only soluble in organic solvents, this study selected maize oil (C116025, Aladdin, Shanghai, China), which is non-toxic to the organism, as the solvent. The low-dose group (L group) was administered at 1/100 of the LD50 (13 mg/kg bw Phe and 13 mg/kg bw Flu), the medium-dose group (M group) at 1/50 of the LD50 (26 mg/kg bw Phe and 26 mg/kg bw Flu), and the high-dose group (H group) at 1/10 of the LD50 (130 mg/kg bw Phe and 130 mg/kg bw Flu) [31,32]. The control group was administered an equivalent frequency and volume of pure corn oil via oral gavage.

Rat body weights were recorded at four-day intervals, with the gavage dosage was adjusted accordingly based on individual body weight. After 60 days of exposure, spontaneously voided urine samples were collected for quantification of Phe and Flu metabolite levels. Subsequently, rats underwent an 8 h fasting period before halothane anesthesia. Cardiac blood was collected, and euthanasia was performed through cervical dislocation. Lung, colon, colonic content, and other organ tissues samples were collected and stored at −80 °C for subsequent biomarker analysis. The experimental procedures were approved by the School of Public Health at Lanzhou University’s IACUC (Protocol No. IRB23061401).

### 2.2. Histopathological Observation of Lung and Colon Tissues

Tissue samples from the lungs and colon were fixed in 4% paraformaldehyde, then embedded in paraffin and sliced into sections around 5 μm thick. These sections were stained with hematoxylin and eosin (H&E) and analyzed under a microscope to assess any morphological changes in the pulmonary and colonic tissues.

### 2.3. Measurement of Oxidative Stress Levels in Lung Tissue

The pulmonary parenchyma was irrigated with pre-chilled normal saline, dissected into uniform sections, homogenized, and subsequently subjected to centrifugation. The resulting supernatant was harvested for protein quantification analysis. Commercial assay kits (Nanjing Jiancheng Bioengineering Institute, Nanjing, China) were employed to determine the concentrations of superoxide dismutase (SOD), malondialdehyde (MDA), catalase (CAT), and reduced glutathione (GSH) in the respiratory tissue.

### 2.4. Detection of Inflammatory Factors in Lung Tissue

Enzyme-linked immunosorbent assay (ELISA) was employed to quantify the concentrations of interleukin-6 (IL-6), interleukin-17A (IL-17A), tumor necrosis factor-alpha (TNF-α), and interleukin-10 (IL-10) in lung tissue samples. The standardized protocol involved the addition of lung homogenate supernatants to ELISA microplates (Elabscience, Wuhan, China), followed by incubation with horseradish peroxidase (HRP)-conjugated detection antibodies. The subsequent addition of chromogenic substrate facilitated colorimetric development, and the optical density was measured spectrophotometrically. Analyte concentrations were determined through quantitative analysis using a validated standard curve.

### 2.5. Western Blotting

The experimental procedure entailed the preparation of lung or intestinal tissues for Western blot analysis by rinsing them with phosphate-buffered saline (Boster, Wuhan, China) and lysing them in radioimmunoprecipitation assay (Boster) buffer containing phenylmethyl-sulfonyl fluoride (Boster) and a phosphatase inhibitor cocktail. Protein concentrations were determined via bicinchoninic acid (Thermofisher, Shanghai, China) assay, followed by denaturation of supernatants at 100 °C for 15 min. Protein separation was achieved through sodium dodecyl sulfate-polyacrylamide gel electrophoresis (Solarbio, Beijing, China), with the resolved proteins subsequently transferred onto polyvinylidene difluoride (Millipore, Billerica, MA, USA) membranes. Subsequently, the membranes were blocked with a non-fat milk solution and incubated sequentially with primary and horseradish peroxidase (HRP)-conjugated secondary antibodies. Quantitative analysis of protein bands was performed using ImageJ software (version win64.exe). Specific antibody information is provided in Supplementary Table S1.

### 2.6. Gut Microbiota and 16S rRNA Sequencing

Gut microbiota analysis and 16S rRNA gene sequencing were carried out by Biomarker (Project ID: BMK230918-BP335-ZX01-0101). High-quality genomic DNA was extracted from colonic contents, and sequencing adapter-linked primers were designed to target conserved regions. Subsequent PCR amplification, purification, quantification, and normalization of amplicons were performed to create a sequencing library. Following rigorous quality control, prior to sequencing on the Illumina NovaSeq 6000 platform (Beijing Biomarker Technologies Co., Ltd., Beijing, China). The initial sequencing reads were processed through merging, quality filtering, clustering/denoising, and taxonomic classification, followed by quantitative analysis to determine microbial community composition. Comprehensive bioinformatic analyses were performed, including alpha and beta diversity assessments, species variation analysis, correlation evaluation, and functional prediction to characterize microbial community dynamics.

### 2.7. Untargeted Metabolomics of Lung and Colonic Contents

The untargeted metabolomics profiling of lung and colonic contents was conducted by Biomarker (Project ID: BMK240326-BY272-ZX01-0101). Metabolites serve as essential biomarkers for characterizing organismal phenotypes and elucidating biological processes. The analytical workflow included metabolite extraction, instrumental analysis, followed by comprehensive qualitative and quantitative evaluation. Samples were analysed using a Waters Acquity I-Class PLUS ultra-high-performance liquid chromatography (Milford, MA, USA) system coupled with a Waters Xevo G2-XS QTOF mass spectrometer. Raw data acquisition was performed using MassLynx V4.2 software, with subsequent data processing employing Progenesis QI software (https://www.waters.com/nextgen/us/en/products/informatics-and-software/mass-spectrometry-software/progenesis-qi-software.html, accessed on 3 October 2025). Metabolite identification was achieved through cross-referencing with the Progenesis QI METLIN database, public metabolite databases, and Biomarker’s proprietary reference database. The generated metabolomic dataset facilitated comprehensive metabolite annotation, quality control assessment, as well as functional prediction and mechanistic analysis.

### 2.8. Bioinformatics and Statistical Analysis

Bioinformatics analyses were performed using QIIME (version 2020.6.0) and R software (version 3.6.1) for comprehensive sequencing data processing, including taxonomic classification and α/β-diversity assessments, as well as data visualization. Detailed information on software parameters and reference databases is provided in Supplementary Table S2. SPSS software (version 26.0) was utilized for statistical analyses. Quantitative results presented as mean ± standard deviation (mean ± SD). One-way analysis of variance (ANOVA) was employed for assessing group variances in normally distributed data with homogenous variances, followed by Tukey’s test for pairwise comparisons. For non-normally distributed data or heterogeneous variances, the Kruskal–Wallis H test was employed. A significance level of α = 0.05 was set, with statistical significance defined as *p* < 0.05.

## 3. Results

### 3.1. Effects of Phe and Flu Exposure on the Intestinal Barrier

**Figure 1:**
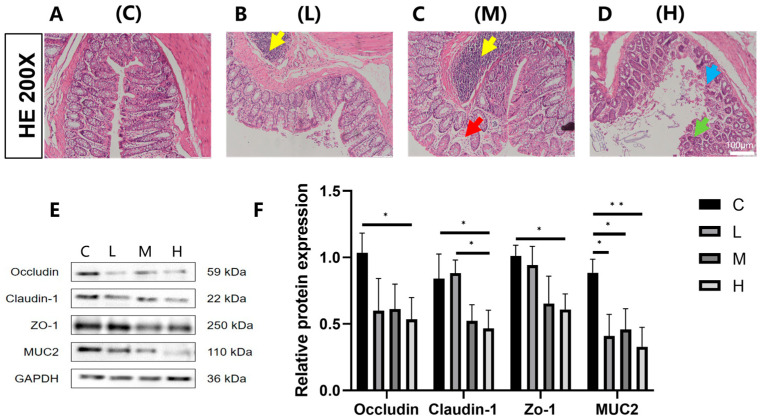
Effects of Phe and Flu exposure on the intestinal barrier: (**A**) colon tissue control group; (**B**) low-dose group of colon tissue; (**C**) medium-dose group of colon tissue; (**D**) high-dose group of colon tissue at 200×. The yellow arrows indicate lymphofollicular hyperplasia, red arrows indicate colonic mucosal edema, blue arrows indicate colonic mucosal erosion, and green arrows indicate abnormal colonic crypt structure and a decrease in the number of goblet cells; (**E**) colonic barrier protein Western blot protein band; (**F**) quantitative analysis of colonic barrier protein bands. Data are presented as mean ± SD (*n* = 3 independent experiments). Statistical significance was determined by one-way ANOVA followed by Tukey’s test, * *p* < 0.05, ** *p* < 0.01.

Following the termination of exposure, HE staining of rat colon tissues (Figure 1A–D) revealed that the control group exhibited colonic architecture, characterized by well-aligned and densely packed epithelial cells. In contrast, with increasing exposure doses, progressive widening of the colonic crypt spacing was observed, accompanied by architectural disruption and reduced crypt depth. Goblet cells, predominantly localized to the crypt bases, demonstrated a marked quantitative reduction. Analysis of tight junction protein expression in colonic tissues (Figure 1E,F) demonstrated that compared to the C group, the L and M exposure groups exhibited a downward trend in occludin, claudin-1, and zonula occludens-1 (ZO-1) protein levels, though these differences failed to reach statistical significance (*p* > 0.05). Notably, the H exposure group manifested statistically significant reductions in occludin, claudin-1, and ZO-1 expression (*p* < 0.05). Furthermore, all exposure dose groups showed significant decreases in mucin 2 (MUC2) protein expression relative to the C group (*p* < 0.05). Collectively, these results provide comprehensive evidence at both histological and molecular levels that Phe and Flu exposure possible disrupt the integrity of the colonic epithelial barrier in rats.

### 3.2. Effects of Phe and Flu Exposure on Gut Microbiota

**Figure 2:**
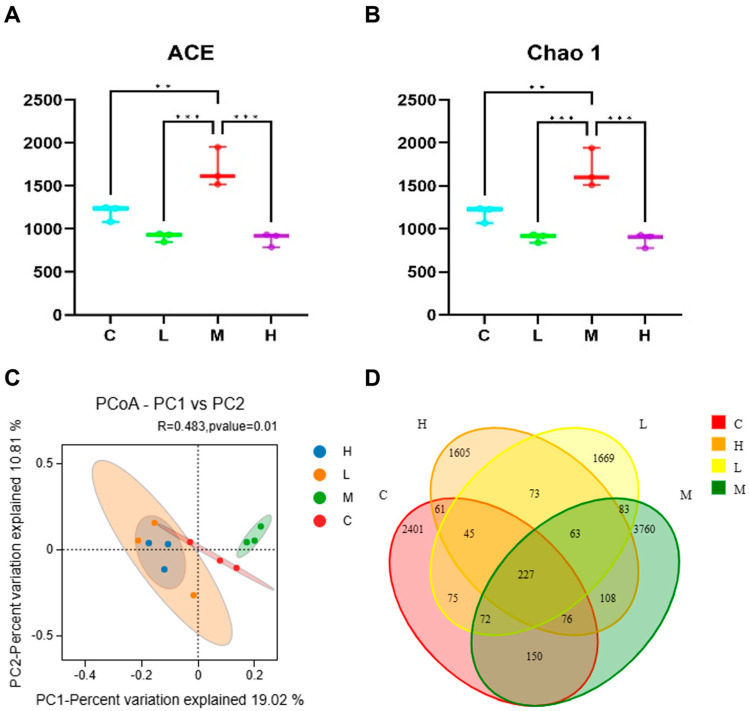
Effects of Phe and Flu infection on intestinal flora: (**A**) ACE index; (**B**) Chao 1 index; (**C**) PCoA analysis; (**D**) the Veen plot shows the difference in OUT levels across all microbial classes between the four groups. Data are presented as mean ± SD (*n* = 3 independent experiments). Statistical significance was determined by one-way ANOVA followed by Tukey’s test for alpha diversity, and PCoA based on Bray–Curtis distance for beta diversity, ** *p* < 0.01, *** *p* < 0.001.

To examine the impact of Phe and Flu exposure on the gut microbiota, 16S rRNA sequencing and subsequent microbial community analysis were conducted on rat colonic contents. Alpha diversity was assessed through the ACE and Chao1 indices. As illustrated in Figure 2A,B, both indices exhibited a decreasing tendency in the low- and high-dose exposure groups relative to the control, although the variations were not statistically significant (*p* > 0.05). Conversely, a significant increase in these indices was observed in the M group (*p* < 0.05). These findings suggest that Phe and Flu exposure led to enhanced diversity of gut microbial communities in the M group rats, whereas the species richness of gut microbial communities was reduced in both the L and H groups. Phe and Flu affected structural changes in the gut microbiota. Additionally, this study employed Principal Coordinates Analysis (PCoA) to explore the β-diversity among different groups. The PCoA analysis of gut microbiota in colonic contents across the four experimental groups (Figure 2C), supplemented with 95% confidence ellipses, revealed that PC1 accounted for 19.02% of the total variance, while PC2 clarified 10.81%. Although partial overlaps were observed among the four groups, their microbial community structures maintained distinguishable characteristics. Notably, the C exhibited clear separation from the L, M, and H treatment groups, demonstrating significant alterations in gut microbiota composition following exposure (*p* < 0.05). The inter-group differences were more pronounced than the intra-group variations. These findings were further substantiated through Venn diagram analysis of operational taxonomic unit (OTU)-level microbial community variations. The intersecting regions denote taxa shared across multiple groups, whereas the non-overlapping segments correspond to group-specific microbial species.

**Figure 3:**
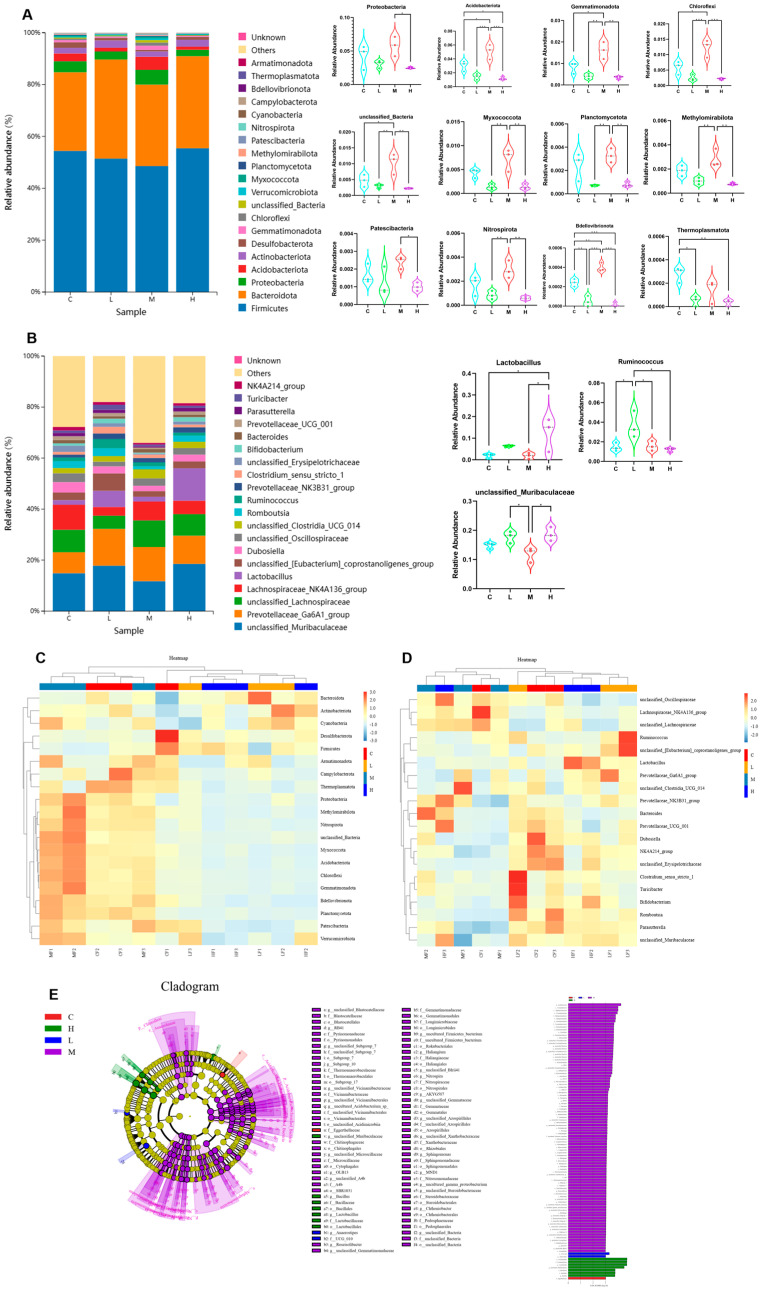
Effects of Phe and Flu infection on microbial composition in intestinal contents: (**A**) stack diagram of the top 20 phylum with the highest species abundance at the phylum level; (**B**) stack map of the top 20 species with the highest species abundance at the genus level; (**C**) heat map of the top 20 phylum with the highest species abundance at the phylum level; (**D**) heat map of the top 20 species with the highest species abundance at the genus level; (**E**) cladogram (left) and LDA score chart (right) with LDA value greater than 3.0 as the screening criterion; statistically significant gut microbial markers were screened out. Data are presented as mean ± SD (*n* = 3 independent experiments). Statistical significance was determined by one-way ANOVA followed by Tukey’s test, * *p* < 0.05, ** *p* < 0.01, *** *p* < 0.001.

The impacts of Phe and Flu exposure on the gut microbiota composition in rats are visualized through stacked bar charts and heatmaps. At the phylum level (Figure 3A,C), the twenty most abundant phyla are displayed, with Firmicutes and Bacteroidetes being the predominant taxa. Compared to the C group, the M group exhibited significant increases in the relative abundances of Acidobacteriota, Gemmatimonadota, Chloroflexi, unclassified Bacteria, Bdellovibrionota, and Thermoplasmatota (*p* < 0.05). Conversely, the H group demonstrated marked downregulation of Acidobacteriota, Bdellovibrionota, and Thermoplasmatota (*p* < 0.05), with notable intergroup differences in phylum-level abundances across dosage groups. Figure 3B,D presents genus-level analyses, revealing a significant upregulation of Lactobacillus in the H group (*p* < 0.05). As a well-established probiotic, Lactobacillus may play a pivotal role in these observed microbial alterations.

Subsequently, for identifying inter-group differences, linear discriminant analysis effect size (LefSe) was performed employing an LDA (linear discriminant analysis) score threshold of >3.0 to determine statistically significant gut microbiota biomarkers. The taxonomic positions of these biomarkers across hierarchical levels were annotated to systematically compare microbial community differences between the C group and the treatment groups (L, M, H) (Figure 3E). The analysis revealed distinct microbial compositions and abundance patterns among the experimental groups. Specifically, the C group exhibited a relatively high abundance of Eggerthellaceae, whereas the L group demonstrated significant differences in UCG 010 and Anaerostipes. The M group displayed the most diverse microbial profile, with marked increases in multiple taxa, including Acidobacteriota, Vicinamibacteria, Alphaproteobacteria, Vicinamibacterales, Sphingomonadaceae, Sphingomonadales, Gemmatimonadota, Vicinamibacteraceae, Gemmatimonadetes, Gemmatimonadales, Chloroflexi, and Gemmationa-daceae. Conversely, the H group was characterized by dominance of Lactobacillales, Lactobacillaceae, and Lactobacillus. These findings suggest that Phe and Flu exposure may contribute to intestinal barrier dysfunction through the interruption of gut microbiota homeostasis.

### 3.3. Effects of Phe and Flu Exposure on Pulmonary Inflammation and Oxidative Stress

**Figure 4:**
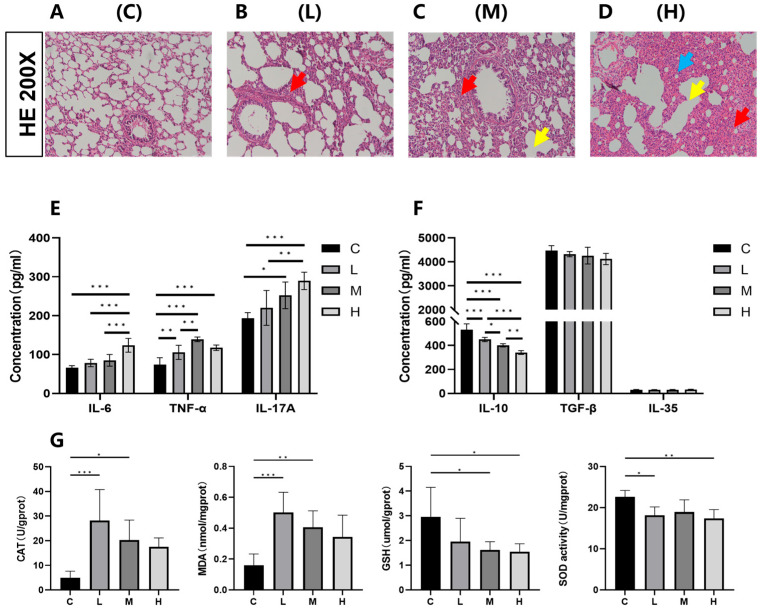
Effects of Phe and Flu on lung inflammatory damage and oxidative stress: (**A**) control group of lung tissue; (**B**) low-dose group of lung tissue; (**C**) medium-dose group of lung tissue; (**D**) high-dose group of lung tissue at 200×. The red arrows indicate alveolar septal congestion, yellow arrows indicate alveolar collapse, and blue arrows indicate inflammatory cell infiltration; (**E**) pulmonary pro-inflammatory factors IL-6, TNF-α, and IL-17A concentration levels; (**F**) pulmonary anti-inflammatory factors IL-10, IL-35, and TGF-β concentration levels; (**G**) pulmonary oxidative stress CAT, MDA, reduced GSH, and SOD concentration levels. Data are presented as mean ± SD (*n* = 6 independent experiments). Statistical significance was determined by one-way ANOVA followed by Tukey’s test, * *p* < 0.05, ** *p* < 0.01, *** *p* < 0.001.

As a distal target organ, histopathological analysis (Figure 4A–D) revealed dose-dependent lung damage in exposed rats compared to the C group, including alveolar exudation, vascular congestion, inflammatory infiltration, alveolar fusion, septal thickening, and epithelial disorganization. These structural abnormalities were corroborated by elevated levels of three pro-inflammatory cytokines—IL-6, TNF-α, and IL-17A (Figure 4E). Compared with the C group, the L group exhibited a significant increase in TNF-α concentration (*p* < 0.05), whereas both the M group and L group showed significant elevations in TNF-α and IL-17A concentrations (*p* < 0.05). The H group demonstrated significant increases in all three pro-inflammatory cytokines (IL-6, TNF-α, and IL-17A) compared to the C group (*p* < 0.05). In the anti-inflammatory factor treatment groups, IL-10 expression levels were significantly reduced across all dosage groups relative to the C group (*p* < 0.05). No statistically significant variation was detected in the expression of the anti-inflammatory cytokines IL-35 and TGF-β across all exposure groups compared with the control group (C) (Figure 4F; *p* > 0.05). Assessment of oxidative stress biomarkers in pulmonary tissue (Figure 4G) revealed that CAT and MDA concentrations were markedly elevated in the low- and medium-dose groups relative to the control (*p* < 0.05), whereas the high-dose group exhibited an upward trend that did not attain statistical significance (*p* > 0.05). Conversely, reduced GSH levels showed a significant decline in the medium- and high-dose groups (*p* < 0.05), while SOD enzymatic activity was notably suppressed in the low- and high-dose groups (*p* < 0.05). These findings collectively indicate that exposure to Phe and Flu induces pulmonary inflammatory damage accompanied by oxidative stress responses.

### 3.4. Effects of Phe and Flu on Lung and Colon Metabolism

**Figure 5:**
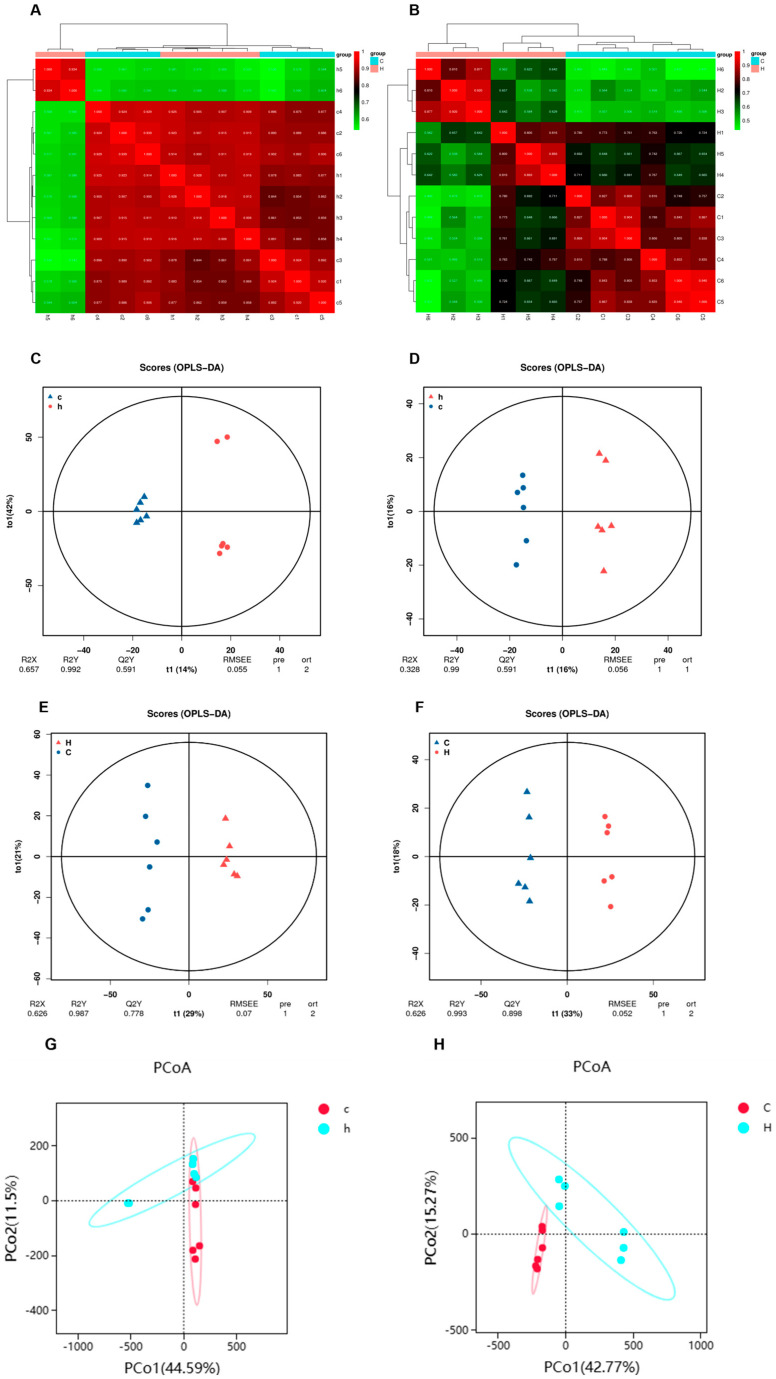
Characterization of the metabolome of lung and colon contents after Phe and Flu infection: (**A**) correlation heat map of lung samples in positive and negative ion mode; (**B**) correlation heat map of colon contents samples in positive and negative ion mode; (**C**) lung OPLS-DA analysis in positive ion mode; (**D**) lung OPLS-DA analysis in negative ion mode; (**E**) OPLS-DA analysis of colon contents in positive ion mode; (**F**) OPLS-DA analysis of colon contents in negative ion mode; (**G**) lung PCoA analysis in positive and negative ion modes; (**H**) PCoA analysis of colonic contents in positive and negative ion mode, *n* = 6 independent experiments.

In evaluating the effects of Phe and Flu on colon and lung tissues, we selected the C and H group samples for untargeted metabolomics. Using LC-QTOF (positive/negative modes), we detected a total of 23,016 ion peaks (4417 annotated metabolites) and identified significant metabolic alterations via multivariate analysis. The quality of intra-group data was assessed by evaluating repeatability through correlation analysis, utilizing the Spearman rank correlation coefficient (r) as the metric for biological repeatability (Figure 5A,B). Intergroup sample differences were evaluated through principal coordinate analysis (PCoA). The PCoA score plot for lung tissue (Figure 5G) demonstrated partial overlap alongside distinct separation between the C group (denoted by red solid ellipse) and the H group (denoted by blue solid ellipse). Notably, in the PCoA score plot for intestinal contents (Figure 5H), complete separation was observed between the C and H groups. To better identify differential metabolites, orthogonal partial least squares discriminant analysis (OPLS-DA) was used to reveal group-specific metabolic trends. This supervised multivariate model reduces noise and enhances discriminative variable extraction, outperforming unsupervised PCoA in classification. In Figure 5C–F, pairwise comparisons between the C and H groups revealed distinct inter-group variations in the OPLS-DA score plots across all experimental groups. Notably, the R^2^Y values approached unity (≈1), while the Q^2^Y values consistently exceeded 0.5, demonstrating robust model repeatability and predictive capability. These findings collectively affirm the high reliability of the experimental dataset.

**Figure 6:**
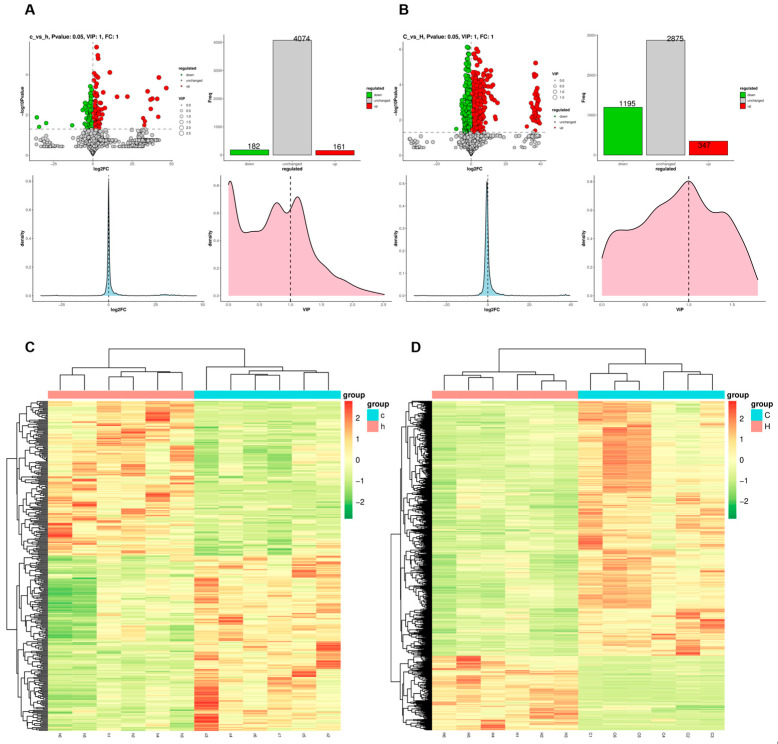
Differences in metabolite disorders in lung and colon contents after Phe and Flu infection: (**A**) StatPlots of differential metabolites in lung tissue in positive and negative ion modes; (**B**) StatPlots of differential metabolites of colon contents in positive and negative ion modes; (**C**) heat map of differential metabolite expression levels in lung tissue in positive and negative ion mode; (**D**) heat map of differential metabolite expression levels of colonic contents in both ionic modes; *n* = 6 independent experiments.

Analysis was conducted in both ionization polarities, employing the screening criteria of VIP > 1, |fold change (FC)| > 1, and *p* < 0.05. A total of 343 differentially expressed metabolites were identified in lung tissue, comprising 161 upregulated and 182 downregulated metabolites (Figure 6A). In colon contents, 1542 differentially expressed metabolites were detected, including 347 upregulated and 1195 downregulated metabolites (Figure 6B). Hierarchical cluster analysis was performed, and the resulting differentially expressed metabolites were visualized as heatmaps (Figure 6C,D), which clearly demonstrated the metabolic disparities between the lung and intestinal tissues of the C and H groups.

**Figure 7:**
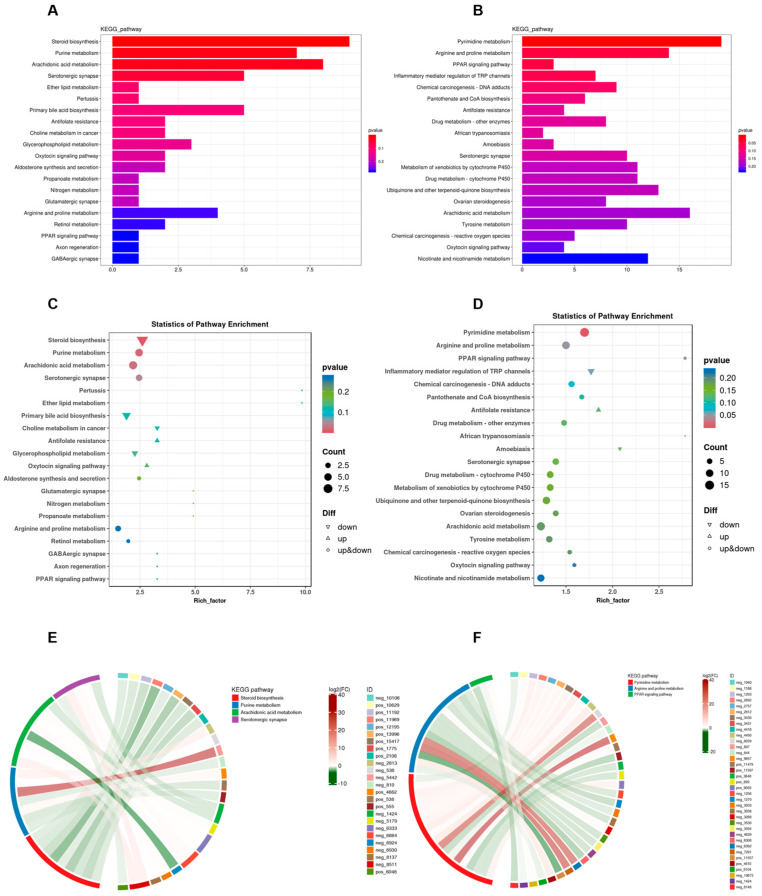
Functional analysis of metabolites in lung and colon contents after Phe and Flu infection: (**A**) histogram of KEGG functional classification of lung differential metabolites; (**B**) KEGG functional classification histogram of differential metabolites in colonic contents; (**C**) scattering plot of KEGG function enrichment of lung differential metabolites; (**D**) scatter plot of KEGG function enrichment of differential metabolites in colonic contents; (**E**) enrichment chord diagram of the correspondence between differential metabolites and metabolic pathways in lung tissue; (**F**) enrichment chord diagram of the correspondence between differential metabolites and metabolic pathways in colonic contents, with red strings representing positive correlation and green strings representing negative correlations. The wider the width of the string, the more counts are associated with this metabolite; *n* = 6 independent experiments.

To elucidate the biological functions of differentially expressed metabolites, this study employed the KEGG database for classification, annotation, and pathway enrichment analysis, thereby identifying key metabolic and signaling pathways associated with these metabolites. As illustrated in Figure 7A–D, the differentially expressed metabolites in lung tissue were predominantly linked with amino acid and lipid metabolism, exhibiting significant enrichment in pathways including steroid biosynthesis, purine metabolism, and arachidonic acid metabolism. In the colon, these metabolites were primarily involved in amino acid metabolism, lipid metabolism, as well as cofactor and vitamin metabolism, with notable enrichment observed in pathways such as pyrimidine metabolism, arginine and proline metabolism, and the PPAR signaling pathway. To further clarify the associations between differentially abundant metabolites and pathways, this study built an enrichment chord diagram (Figure 7E,F), showing that Phe and Flu induce metabolic dysregulation in rat pulmonary and intestinal tissues. These interconnected metabolites form a regulatory network affecting overall metabolic homeostasis.

**Figure 8:**
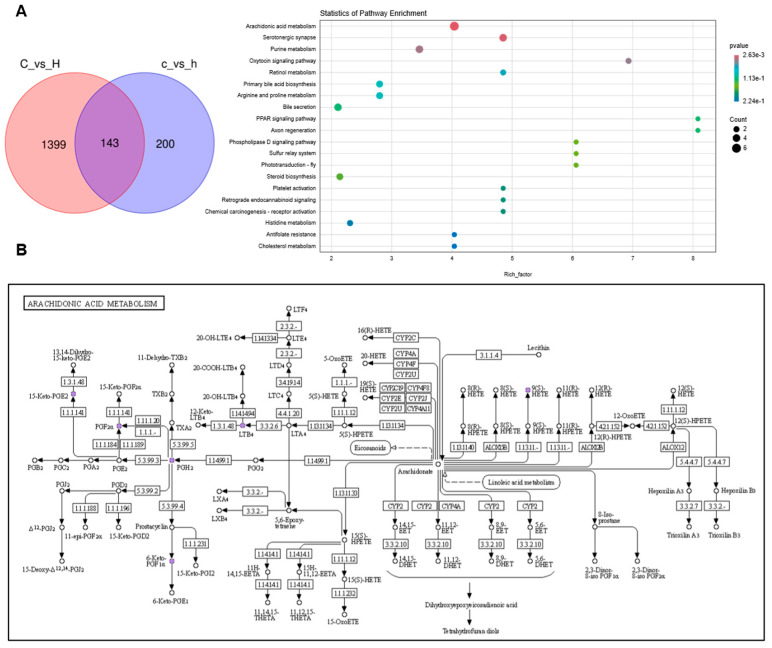

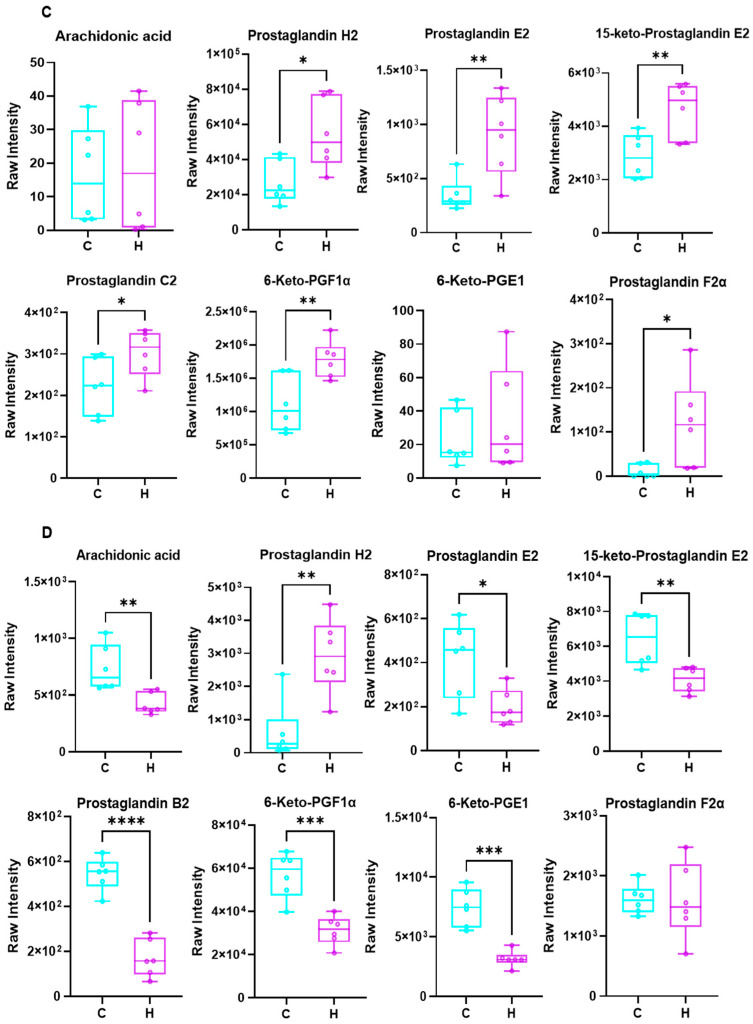
Differential metabolites and KEGG enrichment analysis were performed in lung and colon contents after Phe and Flu infection: (**A**) KEGG enrichment analysis bubble maps were drawn using differential metabolites in the intestine and lung; (**B**) KEGG signaling pathway diagram of AA metabolism; (**C**) relative expression levels of AA metabolites in lung tissue; (**D**) relative abundance of AA metabolites in colonic contents. Data are presented as mean ± SD (*n* = 6 independent biological replicates). Statistical significance was determined by an unpaired two-tailed Student’s *t*-test, * *p* < 0.05, ** *p* < 0.01, *** *p* < 0.001, **** *p* < 0.0001.

Subsequently, a total of 143 differentially expressed metabolites were identified as commonly altered in both lung and colon tissues (Figure 8A). Pathway enrichment analysis revealed that these shared differential metabolites were predominantly involved in AA metabolism, serum neurosynaptic processes, purine metabolism, and the oxytocin signaling pathway. The present investigation primarily focused on AA metabolism, identifying key metabolites comprising AA, prostaglandin H2 (PGH2), prostaglandin E2 (PGE2), prostaglandin C2 (PGC2), and prostaglandin F2α (PGF2α). As illustrated in Figure 8D, relative abundance significant reductions (*p* < 0.05) were observed in the intestinal metabolites AA, PGE2, 15-keto-PGE2, PGB2, 6-Keto-PGF1α, and 6-Keto-PGE1 in the H group relative to the C group. Conversely, as shown in Figure 8C, the lung tissue metabolites PGH2, PGE2, 15-keto-PGE2, PGC2, 6-Keto-PGF1α, and PGF2α exhibited relative abundance significant elevations (*p* < 0.05) in the H group compared with the C group. As is well established, AA metabolism exerts diverse physiological functions in the human body, with its metabolites serving as pro-inflammatory lipid mediators. In conjunction with the metabolomic analyses of lung and colon contents, the present findings indicate that dysregulation in AA may contribute to the pathogenic mechanism underlying pulmonary inflammation.

### 3.5. Joint Analysis Between the Gut Microbiota and Metabolic Profiles

**Figure 9:**
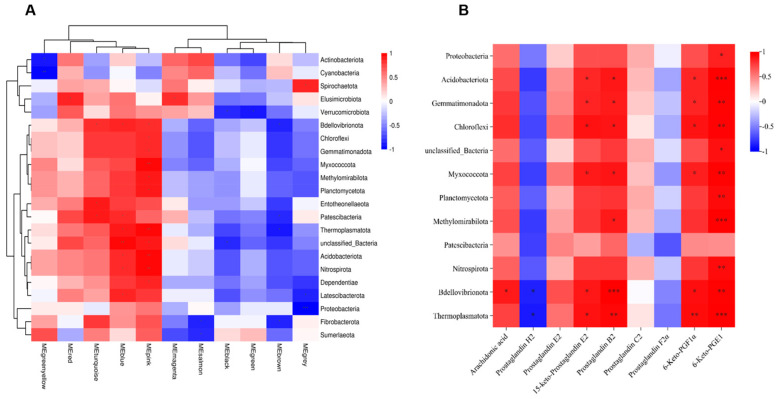
Combined analysis of gut microbiota and metabolites after Phe and Flu exposure: (**A**) metabolite module microbial correlation heatmap; (**B**) heatmap of correlation between AA metabolites and differential phyla, *n* = 6 independent experiments. * *p* < 0.05, ** *p* < 0.01, *** *p* < 0.001.

Joint analysis of gut microbiota and metabolic profiles can reveal underlying biological mechanisms. We used WGCNA on metabolomics data to reduce dimensionality, classify metabolites into modules, and analyze correlations between modules and microbes, noting that individual metabolites/microbes may correlate with multiple others. Therefore, we retained only datasets containing at least one complete correlation matrix, where the combined consistency *p*-values fulfilled the statistical significance threshold (*p* < 0.05). The final dataset was visualized through a heatmap representation. As illustrated in Figure 9A, the metabolites within the MEpink, MEblue, MEturquoise, and MEred modules demonstrated significant positive correlations with the majority of microbial phyla, including Bdellovibrionota, Patescibacteria, and Thermoplasmatota. Conversely, the metabolites in the MEyellow, MEbrown, MEgreen, and MEblack modules exhibited notable negative correlations with predominant microbial phyla, such as Verrucomicrobiota, Thermoplasmatota, and Latescibacterota.

Subsequently, representative AA metabolites and differentially abundant microbes (at the phylum level) were used to construct a heatmap (Figure 9B). The results demonstrated that AA, 15-keto-PGE2, PGB2, 6-Keto-PGF1α, and 6-Keto-PGE1 were significantly positively correlated with certain differentially abundant microbes such as Bdellovibrionota, Thermoplasmatota, Gemmatimonadota, and Acidobacteriota (*p* < 0.05), while PGH2 exhibited significant negative correlations with some of these differential microbes, including Bdellovibrionota and Thermoplasmatota (*p* < 0.05). This finding was consistent with the WGCNA module-microbiota correlation analysis, where metabolites such as AA, 15-keto-PGE2, and 6-Keto-PGF1α were mostly clustered into the turquoise module, while PGH2, PGC2, and PGF2α were clustered into the brown module (Table S3). These findings indicate that the gut microbiota modulate AA metabolism, and, through the gut–lung axis, the resulting metabolites undergo oxidative transformation, subsequently exerting pro-inflammatory effects within the pulmonary tissue.

### 3.6. Metabolites of AA Mediate Pulmonary Inflammation via the Gut–Lung Axis

**Figure 10:**
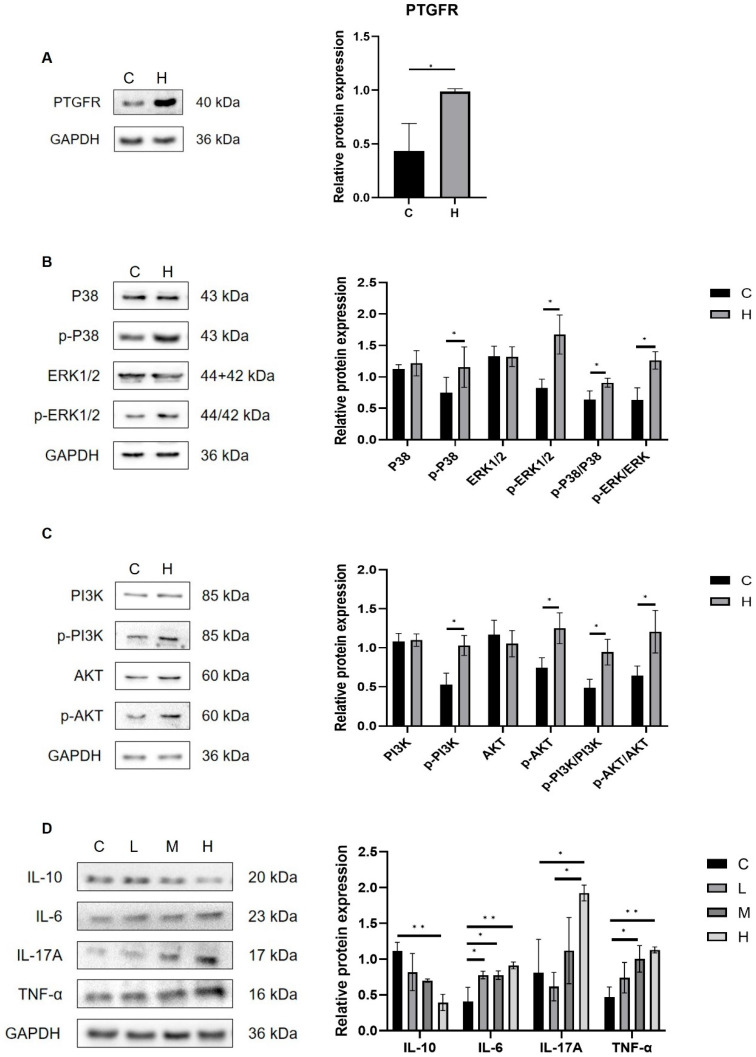
Phe- and Flu-exposed AA metabolites trigger a pulmonary inflammatory response through the gut-pulmonary axis: (**A**) Relative expression level of PTGFR receptor protein; (**B**) relative expression levels of p38, ERK1/2, p-p38, and p-ERK1/2 proteins; (**C**) relative expression levels of PI3K, AKT, p-PI3K, and p-AKT; (**D**) relative expression levels of IL-6, IL-17A, TNF-α, and IL-10 proteins. Data are presented as mean ± SD (*n* = 3 independent biological replicates). Statistical significance was determined by an unpaired two-tailed Student’s *t*-test for comparisons between two groups, and by one-way ANOVA followed by Tukey’s test for comparisons among multiple groups, * *p* < 0.05, ** *p* < 0.01.

Intestinal microbiota and their metabolites influence pulmonary immunity via the gut–lung axis. This study investigated the mechanisms of AA metabolite-related pneumonia by analyzing lung tissue receptor proteins. As illustrated in Figure 10A, the protein expression level of the prostaglandin F receptor (PTGFR) was significantly elevated in the H group relative to the C group (*p* < 0.05). As a homologous member of the G protein-coupled receptor (GPCR) family, PTGFR facilitates the phosphorylation of PI3K/AKT and MAPK/ERK pathways upon binding of AA metabolites to the cell surface FP receptor (PTGFR). This binding subsequently activates the PI3K/AKT and MAPK signaling cascades. Notably, the PI3K/AKT and MAPK/ERK signaling pathways are critically implicated in the pathogenesis of inflammatory diseases. Western blot analysis (Figure 10B,C) demonstrated that, compared with the C group, the lung tissue of the H group exhibited significantly increased protein expression levels of the FP receptor, accompanied by a significant upregulation in the phosphorylated forms of PI3K, AKT, p38, and ERK1/2 (*p* < 0.05). These findings demonstrate that AA metabolites contribute to the progression of pulmonary inflammation by activating the FP receptor-mediated PI3K/AKT and MAPK signaling pathways. Moreover, in the H treatment group, we observed significant upregulation of the pro-inflammatory cytokines IL-6, IL-17A, and TNF-α, accompanied by a marked downregulation of the anti-inflammatory cytokine IL-10 (*p* < 0.05, Figure 10D). Collectively, these results provide further evidence for the critical roles of FP/PI3K/AKT and FP/MAPK signaling cascades in mediating AA metabolite-induced inflammatory injury.

## 4. Discussion

The intestinal mucosa is crucial for nutrient absorption, and structural changes can significantly impair its function. Yu et al. reported that prolonged Phe intake upregulates colitis-associated genes [33]. In the present study, histopathological analysis revealed that Phe and Flu disrupted colonic crypt architecture and decreased goblet cell counts, indicating damage to the colonic epithelium in female rats. Western blot results further demonstrated that Phe and Flu downregulated the expression of Claudin-1, Occludin, ZO-1, and MUC2 proteins. Although these molecular alterations suggest impaired barrier function, direct functional assessments such as dye leakage assays are needed in future work to confirm physiological relevance.

Alterations in gut microbiota composition were also observed, which are known to influence intestinal barrier maturation [34,35]. While no significant differences were found in the relative abundance of Firmicutes and Bacteroidetes or their ratio, exposure to Phe and Flu led to a decrease in potentially beneficial phyla such as Acidobacteriota, which has been linked to improved antioxidant status and intestinal morphology [36,37]. Other less-studied phyla, including Chloroflexi, Bdellovibrionota, and Gemmatimonadota, may also contribute to pathogen resistance and host protection [38,39]. The archaeon Thermoplasmatota, previously reported to correlate negatively with Phe [40], showed reduced abundance under high Phe and Flu exposure. Consistent with Liu et al. [41], these contaminants appeared to exert antibacterial effects, diminishing probiotic abundance and potentially disrupting microbiota homeostasis.

At the genus level, Lactobacillus was significantly increased in the H group. This upregulation may represent a compensatory mechanism whereby the host modulates beneficial bacteria to reinforce mucosal immunity, alleviate inflammation, and mitigate the damage to intestinal barrier integrity caused by Phe and Flu [42]. Our study revealed a notable biphasic effect on gut microbial alpha diversity following exposure to Phe and Flu. Specifically, the M group exhibited a significant increase in diversity, whereas both L and H groups showed a decrease. This pattern is consistent with the concept of hormesis, where a low-level stressor elicits a beneficial adaptive response, while higher levels cause damage [43].

The elevated diversity in the M group may be attributed to the moderate selective pressure exerted by the pollutants, which disrupted the pre-existing community structure without causing overwhelming toxicity. This disruption potentially alleviated competitive exclusion among species, allowing for the co-proliferation of a wider array of bacteria, including stress-tolerant or pollutant-degrading taxa. Conversely, the high-dose exposure likely exceeded the compensatory capacity of the microbial ecosystem, leading to the suppression of sensitive species and a consequent collapse of diversity. Future studies employing metatranscriptomics or targeted cultivation could further elucidate the precise adaptive mechanisms of the gut microbiota under such chemical-induced hormesis.

Both gut microbiota and intestinal barrier integrity are critical to disease development [44]. Often referred to as the “eighth organ,” the gut microbiota influences inflammation and metabolic disorders, and its dysregulation has been shown to modulate signaling pathways that mitigate oxidative stress and lung injury [45,46,47]. This bidirectional gut–lung communication underpins the gut–lung axis [29,48]. In the present study, intestinal barrier disruption by Phe and Flu induced pulmonary inflammatory injury and oxidative stress in female rats. Lung tissue exhibited upregulation of IL-6, TNF-α, and IL-17A, and downregulation of IL-10. Key oxidative stress markers—CAT, SOD, MDA, and reduced GSH—were also altered: CAT and MDA levels increased, while reduced GSH and SOD activities decreased. It is important to note that our assessment of oxidative stress relied on the measurement of reduced GSH levels. While a decrease in reduced GSH is consistent with oxidative stress, we were unable to measure oxidized glutathione (GSSG) and thus could not report the more informative GSH/GSSG ratio. Future studies will specifically include this critical ratio to provide a more comprehensive evaluation of the redox environment.

Growing evidence links gut microbiota composition and barrier function to oxidative stress [49,50]. Correlation analysis (Figure S1) revealed significant microbiota–stress associations: CAT activity negatively correlated with Patescibacteria, while reduced GSH positively correlated with this phylum. Patescibacteria decreased in the H group, suggesting its decline may contribute to increase CAT andGSH. The stabilization of SOD activity in the medium-dose group was positively correlated with the enrichment of a functionally specialized microbial consortium—including Acidobacteriota, Myxococcota, Methylomirabilota, and Thermoplasmatota—which was depleted in high-dose exposed groups. This consortium, characterized by oligotrophic strategists and metabolic specialists, likely enhanced community resilience under chemical stress. Specifically, the enrichment of predatory and secondary degraders like Myxococcota and anaerobes like Methylomirabilota suggests active involvement in xenobiotic processing and ecological redox balancing. We propose that this microbial network attenuated the systemic oxidative burden by mitigating pollutant bioavailability, thereby stabilizing pulmonary SOD activity and underscoring the gut microbiota’s significant role in mediating distant oxidative and inflammatory responses to Phe and Flu exposure.

Metabolites serve as crucial mediators in gut-microbiota communication. Exposure to PAHs disrupts symbiotic microbial communities, thereby interfering with signaling pathways involving purine metabolism, pyrimidine metabolites, and lipid metabolism [51]. As a key PUFA, AA and its metabolites bind to receptors, regulate cellular functions such as apoptosis and growth, and are associated with chronic diseases [52]. Studies indicate that AA and its metabolites can alter gut microbiota composition and trigger systemic low-grade inflammation [53,54]. This study revealed a significant correlation between six AA metabolites and eight bacterial phyla. Exposure to Phe and Flu disrupted AA metabolism, leading to decreased levels of AA and E/F series PGs in the colon, while promoting their accumulation in the lungs. This pulmonary accumulation of E/F series PGs induced lung tissue inflammation by recruiting leukocytes from systemic circulation, thereby shifting the tissue microenvironment toward a pro-inflammatory state [55]. While the relative changes in AA metabolites reported here are based on a rigorously controlled metabolomics platform and are consistent with the inflammatory phenotype, future studies will include targeted, absolute quantification of these key mediators to confirm their precise levels.

The findings reveal that dietary intake of PAHs can induce DNA adducts in the lungs, and translocation of toxic metabolites between organs may produce “long-distance” systemic effects [20]. In this study, the pulmonary effects induced by Phe and Flu exposure in the colon manifested as characteristic “long-distance” systemic manifestations. Accumulation of AA and its metabolites in lung tissue via the gut–lung axis activates PI3K/AKT and MAPK signaling cascades, upregulating pro-inflammatory mediators and downregulating anti-inflammatory factors. This study highlights PGs metabolites PGF2α and PGF1α, which interact with FP receptors to modulate inflammatory pathways [56]. PGF1α and PGF2α activate PI3K, ERK1/2, and p38 MAPK signaling cascades [57,58,59]. These pathways are crucial in regulating oxidative stress and inflammation. Elevated levels of PGF1α and PGF2α in lung tissues, along with FP upregulation, lead to increased phosphorylation of p38, PI3K, ERK1/2, and AKT, activating MAPK and PI3K/AKT pathways. This activation results in the production of inflammatory mediators, including IL-6, IL-17A, and TNF-α. The FP/MAPK and FP/PI3K/AKT pathways are key in PG-induced lung inflammation and oxidative stress responses. Studies show that FP inhibition reduces LPS-induced neutrophil infiltration and downregulates pro-inflammatory cytokines [60].

Therefore, this study provides systematic evidence that Phe and Flu induce lipid metabolism dysregulation by compromising intestinal barrier integrity and disrupting gut microbial homeostasis, thereby facilitating the translocation of inflammatory mediators to the lungs through the gut–lung axis. These findings further elucidate that PGs trigger pulmonary oxidative stress and inflammatory responses via activation of the FP receptor-mediated MAPK and PI3K/AKT signaling pathways.

## 5. Limitations

This study has several limitations. The functional roles of the identified discriminant gut microbiota and metabolites in the toxicological process remain to be experimentally validated through approaches such as gnotobiotic models or metabolite supplementation. Furthermore, the exclusive use of female rats, while aligning with our initial epidemiological focus, limits the generalizability of our findings across sexes, especially given the known sexual dimorphism in immune and metabolic responses. Finally, the mechanistic links within the gut–lung axis, particularly the involvement of the AA pathway, are inferred from multi-omics correlations and the literature; thus, causality awaits confirmation via complementary in vitro assays. Notwithstanding these limitations, our findings provide a valuable correlative foundation and generate specific, testable hypotheses for future research.

## 6. Conclusions

Exposure to LMW-PAHs (Phe and Flu) induces pulmonary inflammation and oxidative stress through the gut–lung axis. This process is initiated by dysregulation of the gut microbiota, which alters the intestinal metabolic profile and reduces the abundance of specific intestinal prostaglandin E and F metabolites. These metabolites are subsequently transported to the pulmonary system through the gut–lung axis, where they trigger the activation of FP/MAPK and FP/PI3K/AKT signaling pathways within lung tissue, ultimately contributing to the regulation of inflammatory reactions and oxidative stress in the lungs.

## Data Availability

The original contributions presented in this study are included in the article/Supplementary Materials. Further inquiries can be directed to the corresponding authors.

## Supplementary Materials

The following supporting information can be downloaded at: https://www.mdpi.com/article/10.3390/toxics13121017/s1, Table S1. All Antibody details. Table S2. Relevant software parameters and databases. Table S3. Partial metabolite WGCNA module. Figure S1. Correlation matrix diagram of microorganisms and oxidative stress biomarkers.
