# Supplementary material for: Involvement of the Gut–Lung Axis in LMW-PAHs-Induced Pulmonary Inflammation
Source: Toxics. 2025 Nov 25;13(12):1017. doi: 10.3390/toxics13121017 (PMC12737593; doi:10.3390/toxics13121017)
Supplement: Supplementary file 1 [file toxics-13-01017-s001.zip › toxics-3938858-supplementary.pdf]

# Supplementary Datas

Table S1. All Antibody details

| Antibody | Product code | Producer |
| --- | --- | --- |
| Claudin-1 Antibody | 29226 | Signalway Antibody LLC |
| Occludin Antibody | 29275 | Signalway Antibody LLC |
| ZO-1 Antibody | 29274 | Signalway Antibody LLC |
| MUC2 Antibody | 53898 | Signalway Antibody LLC |
| PTGFR | 28822 | Signalway Antibody LLC |
| P38 | 14064-1-AP | Proteintech Group |
| ERK1/2 | ab184699 | Abcam |
| p-P38 | 28796-1-AP | Proteintech Group |
| p-ERK1/2 | 12548 | Signalway Antibody LLC |
| PI3K | ab226826 | Abcam |
| AKT | 21055 | Signalway Antibody LLC |
| p-PI3K | ab235266 | Abcam |
| p-AKT | ab38449 | Abcam |
| IL-6 | 41739 | Signalway Antibody LLC |
| IL-17A | 38147 | Signalway Antibody LLC |
| TNF-α | 41504 | Signalway Antibody LLC |
| IL-10 | 41553 | Signalway Antibody LLC |
| GAPDH | 37985 | Signalway Antibody LLC |
| Goat anti-Rabbit IgG Secondary Antibody HRP | L3012 | Signalway Antibody LLC |

Table S2. Relevant software parameters and databases

| Software | Version | Analysis_module |
| --- | --- | --- |
| rimmomatic | 0.33 | Data process |
| cutadapt | 1.9.1 | Data process |
| usearch | 10.0.240_i86 | Data process |
| FASTX Toolkit | 0.0.14 | Data process |
| flash | 1.2.11 | Data process |
| fastp | 0.23.1 | Data process |
| QIIME2 | 2020.6.0 | -- |
| dada2 | 1.20.0 | Denoise |
| lefse | 1.1.1 | Difference analysis |
| R | 3.6.1 | Analyze the locale as a whole |
| prcomp(R base function) 3.6.1 | 3.6.1 | PCA |
| pheatmap | 1.0.2 | Heatmap |

| | | |
|---|---|---|
| ropls | 1.6.2 | OPLS-DA analysis |
| corrplot | 0.73 | Correlation heat map |
| clusterProfiler, enrichplot | 4.4.4, 1.2.0 | Enrichment analysis software |
| friend | 1.6 | Venn diagram |

| Database | Description | Home page |
|---|---|---|
| KEGG | The database of Kyoto Encyclopedia of Genes and Genomes | http://www.genome.jp/kegg/ |
| HMDB | HMDB: the Human Metabolome Database | https://hmdb.ca/ |
| Lipidmaps | The LIPID MAPS Structure Database (LMSD) is a relational database encompassing structures and annotations of biologically relevant lipids | https://lipidmaps.org/ |

Table S3. Partial metabolite WGCNA module

| name | Module | name | Module |
|---|---|---|---|
| UDP-2,4-bis(acetamido)-2,4,6-trideoxy-beta-L-altropyranose | blue | 19-Hydroxytestosterone | brown |
| (R)-3-Amino-2-methylpropanoate | blue | Spheroidenone | brown |
| 2-Hydroxyglutaric acid lactone | blue | Dihydroceramide | brown |
| PG(i-19:0/PGF1alpha) | blue | Prostaglandin C2 | brown |
| PE(P-16:0/PGF1alpha) | blue | PG(a-17:0/PGF2alpha) | brown |
| 2,3-Dinor-11b-PGF2α | blue | Prostaglandin H2 | brown |
| PGF1α | blue | Ganoderenic acid C | brown |
| CDP-DG(PGF2alpha/α-17:0) | blue | Pangamic acid | brown |
| 7-Hydroxy granisetron | blue | Prostaglandin F2alpha | brown |
| Propylene carbonate | blue | Malvalic acid | brown |
| 21-Hydroxyisoglabrolide | blue | Atraric acid | brown |
| N-Palmitoyl Lysine | blue | Choline | brown |
| Pentadecanal | blue | Miglustat | brown |
| E-64 | blue | Traumatin | brown |
| Etoposide | blue | Urocortisol | brown |

| | | | |
|---|---|---|---|
| Alamandine | blue | Norlaudanosoline | brown |
| HU210 | blue | Urobilinogen | brown |
| Calindol | blue | Mepartricin | brown |
| Curcumenone | blue | L-Thyronine | brown |
| Gossypol | blue | Glycitein | brown |
| Lanosterol | green | Sclareol | grey |
| Campestanol | green | CID 9844747 | grey |
| PA(13:0/5-iso PGF2VI) | green | TG(10:0/18:0/8:0) | grey |
| Polyporusterone B | green | Pent-1-en-2-ol | grey |
| Cichorioside D | green | MG(0:0/14:0/0:0) | grey |
| Oryzalexin B | green | Sorbitan laurate | grey |
| 4,6-Tricosanedione | green | Indoxyl sulfate | grey |
| DG(PGF2alpha/2:0/0:0) | green | 5-Phosphooxy-L-lysine | grey |
| Brassinolide | green | Flurandrenolide | grey |
| SM(d16:1/PGF2alpha) | green | Tenofovir exalidex | grey |
| PG(18:1(11Z)/TXB2) | green | PC(P-16:0/PGE1) | grey |
| 13,14-dihydro-15-keto-PGF2alpha | green | 14-Demethyllanosterol | grey |
| Adynerin | green | Isopentenyl phosphate | grey |
| Arachidonoyl Serinol | green | LysoPE(P-18:1(9Z)/0:0) | grey |
| Ganoderic acid delta | green | Dihydrobiopterin | grey |
| Tangeraxanthin | green | O-Ureido-L-serine | grey |
| Penitrem D | green | 7,8-Diaminononanoate | grey |
| Maslinic Acid | green | L-Anticapsin | grey |

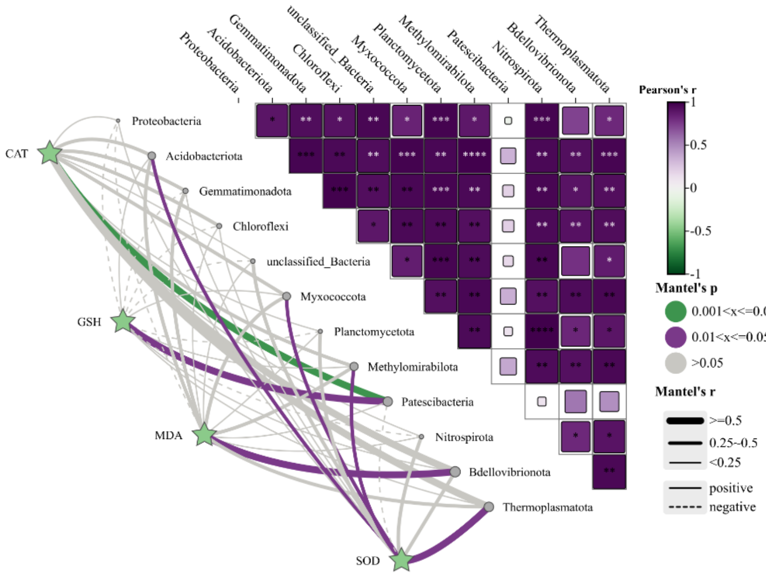


Figure S1. Correlation matrix diagram of microorganisms and oxidative stress biomarkers. *$p$<0.05,**$p$<0.01, ***$p$<0.001.
